# Genetic and epigenetic architecture of paternal origin contribute to gestation length in cattle

**DOI:** 10.1038/s42003-019-0341-6

**Published:** 2019-03-14

**Authors:** Lingzhao Fang, Jicai Jiang, Bingjie Li, Yang Zhou, Ellen Freebern, Paul M. Vanraden, John B. Cole, George E. Liu, Li Ma

**Affiliations:** 1 Department of Animal and Avian Sciences, University of Maryland, College Park, MD 20742 USA; 2 Animal Genomics and Improvement Laboratory, BARC, Agricultural Research Service, USDA, Beltsville, MD 20705 USA; 3 Key Laboratory of Agricultural Animal Genetics, Breeding and Reproduction, Education Ministry of China, Huazhong Agricultural University, 430070 Wuhan, Hubei China

## Abstract

The length of gestation can affect offspring health and performance. Both maternal and fetal effects contribute to gestation length; however, paternal contributions to gestation length remain elusive. Using genome-wide association study (GWAS) in 27,214 Holstein bulls with millions of gestation records, here we identify nine paternal genomic loci associated with cattle gestation length. We demonstrate that these GWAS signals are enriched in pathways relevant to embryonic development, and in differentially methylated regions between sperm samples with long and short gestation length. We reveal that gestation length shares genetic and epigenetic architecture in sperm with calving ability, body depth, and conception rate. While several candidate genes are detected in our fine-mapping analysis, we provide evidence indicating *ZNF613* as a promising candidate for cattle gestation length. Collectively, our findings support that the paternal genome and epigenome can impact gestation length potentially through regulation of the embryonic development.

## Abstract (web summary)

Lingzhao Fang et al. studied the paternal genetic variants that affect gestational length in cattle. They found that paternal genes from pathways involved in embryonic development were associated with gestation length, and that these were often found in differentially methylated regions of the genome.

## Introduction

Gestation length measures the fetal development period from conception to subsequent parturition in mammals, which is crucial for mammalian development. The events occurred in gestation period can have important consequences for the health, productivity, and fertility of the offspring^1^. The abnormality of gestation length can lead to either preterm or post-term birth, resulting in acute and long-term adverse health outcomes in humans^2–4^. In cattle, the length of gestation highly correlates with health, production, and reproduction performances^5^. For instance, prolonged gestation length has been reported to be associated with increased fetal weight, reduced pregnancy rate, and more difficult calving in dairy cattle^5,6^. Furthermore, gestation is a unique immunological state (i.e., the immune clock of pregnancy) that may help predict preterm birth (i.e., shortened gestation length)^7^ that can influence the risk of developing immune-related diseases^8^. Moreover, gestation length as a trait also has direct applications to the dairy industry, because more precise expected dates can be used to assist herd management practices on health and nutritional aspects^5^.

Gestation length is a complex phenotype affected by many genetic and environmental factors, including the progesterone rise, prenatal growth, maternal age, and maternal and fetal immune systems^9–11^. It has been reported that gestation length is a highly heritable trait with a heritability of 30–50% in humans and cattle^10,12,13^. By measuring properties of pregnancy, gestation length also has a complex genetic architecture with direct contributions from maternal and fetal genomes, and likely an indirect, paternal influence through regulation of the fetal development. Many studies in humans have explored the maternal genetic factors that were associated with gestation length^4,14,15^, but few of them have investigated the indirect impacts of paternal genetics on gestation length, possibly due to the limited availability of data.

The U.S. dairy industry has a long history of collecting phenotypic records of dairy cattle. Based on millions of mating and dairy production records, the U.S. dairy cattle database has archived a large amount of reliable phenotypes on gestation length for thousands of service bulls. This data resource provides valuable information to study the paternal impacts on gestation length in mammals. A previous study reported that the heritability of gestation length for service sires (the paternal contribution) was about 47 and 33% when mated to heifers and cows, respectively^10^. On the other hand, the epigenetic information in sperm has been shown to influence the embryonic development through regulating gene expression in embryos^16–18^, warranting interest to explore whether the epigenetic alterations in sperm can also contribute to gestation length.

In this study, we seek to investigate the paternal genetic contribution to gestation length in cattle through associating ~3 million imputed sequence variants with gestation length in a large sample of 27,214 Holstein bulls. In addition, we characterized genome-wide DNA methylation alterations in sperm that were associated with gestation length and three genetically correlated traits of economic importance, sire calving ease (SCE), body depth (BDE), and cow conception rate (CCR), by comparing sperm methylomes of 18 representative animals with extreme phenotypes^19^. Moreover, we integrated the genetic variants of gestation length with DNA methylation alterations in sperm. We further validated our findings by examining publicly available transcriptome data across 87 adult and embryonic tissues^20^. Finally, we provided genetic, epigenetic, and selection evidence implicating a candidate gene *ZNF613* for gestation length. Collectively, our results illustrated the importance of paternal genome and epigenome to gestation length through regulation of embryonic development, and provided insights into the genetic and biological mechanisms underpinning gestation length. We believe that our findings in cattle can provide valuable knowledge for other mammals, including human and rodents.

## Results

### The complex genetic architecture underlying gestation length

**Fig. 1:**
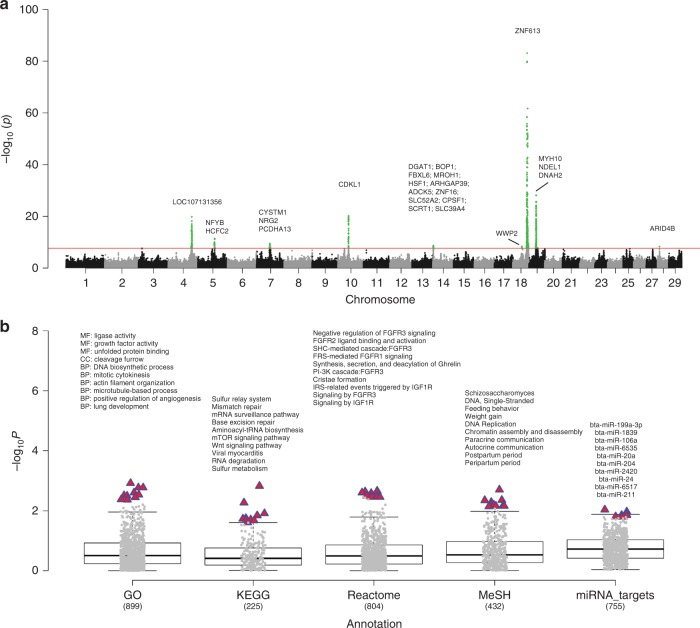
Single-marker GWAS, gene-level fine-mapping, and GWAS signal enrichment of gestation length. **a** Manhattan plot of all the imputed sequence variants being tested. Red line denotes the genome-wide significance of *P* equals to 1.91e-08. All the candidate genes are determined with the posterior probability of causality >0.05 based on gene-level fine mapping (BFMAP). **b** GWAS signal enrichment on the basis of five gene annotation sources, including Gene Ontology (GO), Kyoto Encyclopedia of Genes and Genomes (KEGG) pathway, Reactome metabolism pathway, Medical Subject Headings (MeSH), and miRNA-target networks. The values at x axis are the number of genomic features (i.e., gene lists) being tested in the corresponding annotation sources. The red triangle denotes the top 10 items with the highest enrichments (i.e., −log_10_*P*) in each annotation source. The names of these items are shown in the figure

A total of 27,214 Holstein bulls with ~3 million imputed sequence variants and highly reliable phenotypes were included in the current analyses. In total, our single-marker GWAS revealed nine quantitative trait loci (QTL) that were located in the *Bos taurus* chromosome (BTA) 4, 5, 7, 10, 14, 18, 19, and 28, respectively (Fig. 1a). BTA 18 had two QTLs with one of them being the most significant, consistent with a previous study that used a smaller sample size of 4743 bulls^21^. Our following fine-mapping analyses on these nine QTL regions determined 25 candidate genes (posterior probability of causality > 0.05) for gestation length, including multiple genes participating in the embryonic development (e.g., *HSF1*^22^, *MYH10*^23,24^, *NDEL1*^25^, and *NRG2*^26^), immune responses (e.g., *HCFC2*, and *CYSTM1*), DNA processing (e.g., *WWP2*, *CDKL1*, *ZNF613*, and *CPSF1*), and cell differentiation (e.g., *ZNF16* and *ARID4B*) (Fig. 1a; Supplementary Data 1). By examining the Bovine Gene Atlas data that measured the transcriptome across 87 tissues in cattle (http://www.innatedb.com/)^20^, we found that 5 out of 25 fine-mapped genes exhibited the highest expression level in placenta, including *SLC39A4*, *WWP2*, *DNAH2*, *ZNF613,* and *MYH10*. We further found five fine-mapped genes having the highest expression in the immune and growth-related glands (e.g., thymus, anterior pituitary and thyroid), including *NFBY*, *NRG2*, *FBXL6*, *ZNF613*, and *ARID4B*, among which *NFBY* and *ZNF613* were also highly expressed in the embryonic tissues (i.e., fetal tongue surface) (Supplementary Data 1). Through examining the human GWAS Catalog (https://www.ebi.ac.uk/gwas/home), we found that the human orthologues of three fine-mapped genes, *DNAH2*, *CYSTM1*, and *WWP2*, have been reported to be significantly associated with vascular endothelial growth factor levels, intelligence (neurogenesis and myelination), and menarche in human, respectively, suggesting their important roles in development and fertility (Supplementary Data 1). Together, all these results suggest that our fine-mapped genes likely affect gestation in a tissue-specific manner and potentially through the regulation of the fetal genome and development.

Based on the omnigenetic model of complex phenotypes^27^, we conducted GWAS signal enrichment analyses to determine the core molecular interaction networks that are engaged in regulating gestation length. We employed five commonly used gene annotation sources, including Gene ontology (GO), Kyoto Encyclopedia of genes and genomes (KEGG) pathway, Reactome metabolism pathway, Medical subject headings (MeSH), and miRNA-target networks (miRBase). As shown in Fig. 1b (See Supplementary Data 2 for details), various biological processes and pathways may affect gestation length, including multiple embryonic developmental processes (e.g., positive regulation of angiogenesis, Wnt signaling pathway, mTOR signaling pathway, and mRNA surveillance pathway), growth factors signaling pathways (e.g., fibroblast growth factor receptors (FGFRs), ghrelin synthesis and insulin-like growth factor 1 receptor (IGF1R) regulation), and DNA processing pathways (e.g., DNA biosynthetic process, DNA replication and mismatch repair). These results were consistent with previous findings that DNA damage was repaired very effectively during pregnancy in humans^28^. Analyses on the basis of MeSH revealed that gestation length was strongly associated with feeding behaviors, postpartum and peripartum periods, and autocrine and paracrine communications, in agreement with a previous study that reported the important roles of autocrine and paracrine signaling in the embryonic skeletal development through regulating the IGF1R signaling pathways^29^. Our miRNA-target network analyses showed that 84 out of the 755 tested miRNAs were significantly (*P* < 0.05) involved in gestation length (Supplementary Data 2), and their targets were enriched (FDR < 0.05) in the regulation of action cytoskeleton, miRNA surveillance pathways, multiple growth-related hormone metabolism (e.g., adrenergic signaling and parathyroid hormone synthesis, secretion and action), and immune responses (e.g., leukocyte transendothelial migration and bacterial invasion of epithelial cells) (Supplementary Fig. 1). Of note were the enrichments of all the miRNA-targets, which were significantly (*P* < 0.01; *t*-test) higher than all the other four annotation databases (Fig. 1b; Supplementary Data 2). Previous studies showed that maternal plasma miRNAs can be used as a biomarker during pregnancy to predict preterm birth and pregnancy loss in human^30,31^, and the altered expression of circulating miRNAs has also been proposed to be associated with pregnancy in cattle^32^. We further validated that 8 and 4 out of the 84 significant miRNAs were differentially expressed in milk^33^ and plasma^34^, respectively, during early pregnancy in cattle, including bta-miR-20a, bta-miR-106b, bta-miR-100, bta-miR-143, bta-miR-99b, bta-miR-125b, bta-miR-125a, bta-miR-93, bta-miR-99a-5p, bta-miR-99b, bta-miR-125b, and bta-miR-29a. Our findings were consistent with previous findings that miRNAs played an important role in pregnancy, especially for fetal growth and regulation of the immune system^35,36^.

**Fig. 2:**
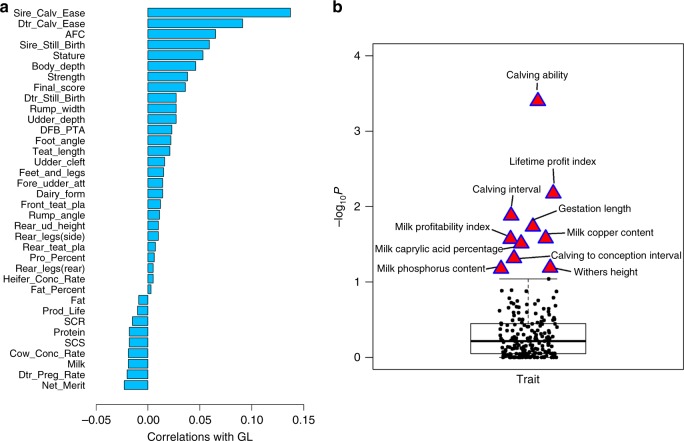
Genetic relationships of gestation length and other dairy traits of economic importance. **a** Genetic correlations between gestation length and 35 dairy traits in the U.S. Holstein population. The genetic correlations are approximately computed using the effects of all tested variants in GWAS. **b** GWAS signal enrichment of gestation length based on the Cattle QTLdb (https://www.animalgenome.org/cgi-bin/QTLdb/BT/index). Each dot denotes a list of genes that are associated with a complex trait in Cattle QTLdb. The top 10 traits with the highest enrichments (i.e., −log_10_*P*) are shown in the figure

Combining gestation length with other economically important dairy traits, we showed that gestation length was significantly positively correlated with calving ability (i.e., service sire and dam effects on still birth and calving ease, which were combined to measure calving ability) and body conformation traits (e.g., stature and body depth), whereas it was significantly, yet negatively correlated with conception/pregnancy rates and milk production performance (Fig. 2a). Through conducting GWAS signal enrichment analysis using QTLs of 208 complex traits in the Cattle QTLdb^37^ (https://www.animalgenome.org/cgi-bin/QTLdb/index), we confirmed that gestation length was not only highly associated with calving ability and withers height, but also highly associated with milk caprylic acid percentage, as well as milk phosphorus and copper content (Fig. 2b; Supplementary Data 2). This was consistent with other studies that indicated phosphorus and copper deficiency can affect prenatal development and pregnancy outcomes in human^38,39^. These results suggest that gestation length may share the underlying genetic basis with many dairy traits of economic importance, reflecting the biological and genetic complexity as well as economic implications of gestation length in cattle.

### Sperm methylation changes associated with gestation length

**Fig. 3:**
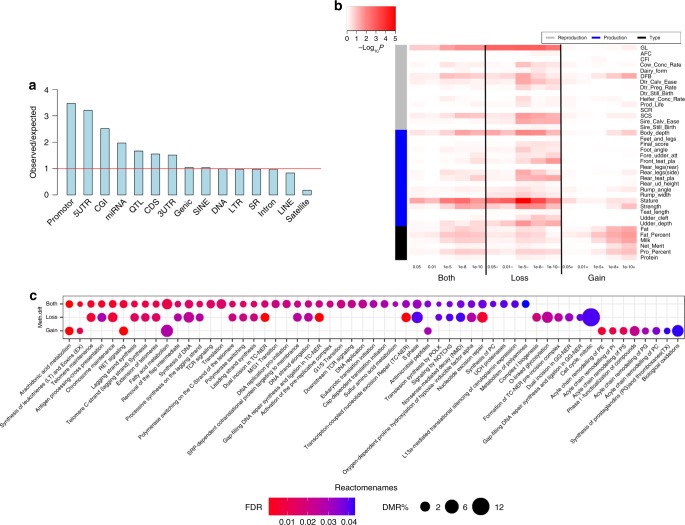
Sperm DNA methylation alterations associated with gestation length. **a** Enrichment of differentially methylated regions (DMRs) across multiple genomic elements. The enrichment of DMRs on a genomic element is computed as the ratio between the observed density of DMRs in this particular genomic element and the expected density of DMRs in the entire genome. **b** GWAS signal enrichment of gestation length based on DMRs that were determined by comparing animals with high gestation length to those with low gestation length. DMRs are first defined based on five different *q* values cutoffs (i.e., 0.05, 0.01, 1e-5, 1e-8, and 1e-10) and the absolute value of methylation difference >5% (Both). DMRs were then divided into two subsets according to the sign of methylation difference, i.e., <−5% (Loss) and > 5% (Gain). **c** DMR-set enrichment analysis based on the Reactome pathway database

By comparing sperm methylomes between bulls with high and low gestation length, we aimed to determine the differentially methylated regions (DMR) in sperm (adjusted *P* value (*q*) < 0.01 and the absolute difference in methylation >5%), which may contribute to gestation length through regulating the fetal development. In total, we detected 66,318 out of 593,035 tested regions as significant DMRs, and a QQ plot for the DMR analysis was shown in Supplementary Fig. 2. We found that many of these gestation length associated DMRs were clustered in BTA 13, 18, and 25 as compared to other chromosomes (Supplementary Fig. 3). We further observed that gestation length associated DMRs intersected many genomic elements, but were more likely to be enriched in promoters, CpG islands, miRNAs, and QTLs of gestation length (i.e., ±1 Mb around the top associated SNPs) (Fig. 3a). Since there was an inflation of the test statistics in the QQ plot of the DMR analysis, our analysis was not focused on the significant DMRs. Instead, we defined multiple sets of DMRs with different *q* value cutoffs, and then investigated the enrichment of GWAS signals in each DMR set using GWAS signal enrichment analysis (see Methods). Our results revealed that GWAS signals were significantly (*P* < 0.05) enriched in gestation length associated DMRs. This enrichment was also significant for other traits, including days to first breeding after calving (DFB), somatic cell sore (SCS, which is highly related to mastitis^40^), multiple body type traits (e.g., strength and stature), and milk production traits (e.g., milk and fat yields). Of note, we found that DMRs that lost methylation in animals with higher gestation length had stronger enrichment (i.e., smaller *P*-value) than those that gained methylation across gestation length and multiple reproduction and body type traits (Fig. 3b). However, milk production traits exhibited an opposite trend, where DMRs that gained methylation in animals with higher gestation length had more significant enrichment than those that lost methylation (Fig. 3b). Our DMR-set enrichment using Reactome pathways further validated that DMRs that lost methylation in animals with higher gestation length were significantly enriched in pathways related to telomere and chromosome maintenance, translation, and DNA damage repairs, suggesting their potential role in pregnancy and embryonic development^41,42^ (Fig. 3c; Supplementary Data 3). DMRs that gained methylation were significantly engaged in lipid metabolism (e.g., arachidonic acid metabolism and fatty acid metabolism), indicating their important roles in the regulation of milk production (Fig. 3c; Supplementary Data 3). Furthermore, we found that 17 out of the 25 fine-mapped genes were overlapped (gene body and promoter) with DMRs (Supplementary Data 4), and eight of them were reported to be transcriptionally active during early embryonic development (from four cells to blastocyst) before implantation^43^, including *ARID4B*, *HCFC2*, *NFYB*, *CPSF1*, *WWP2*, *ZNF613*, *NDEL1,* and *PCDHA13* (Supplementary Data 4). All of these results provide evidence that sperm methylation alterations influence gestation length through regulating fetal development, as well as suggest that epigenetic alterations in germline cells induced by environmental perturbations may impact complex phenotypes through a transgenerational inheritance model^44^, potentially contributing to the genetic architecture underlying complex traits and diseases.

**Fig. 4:**
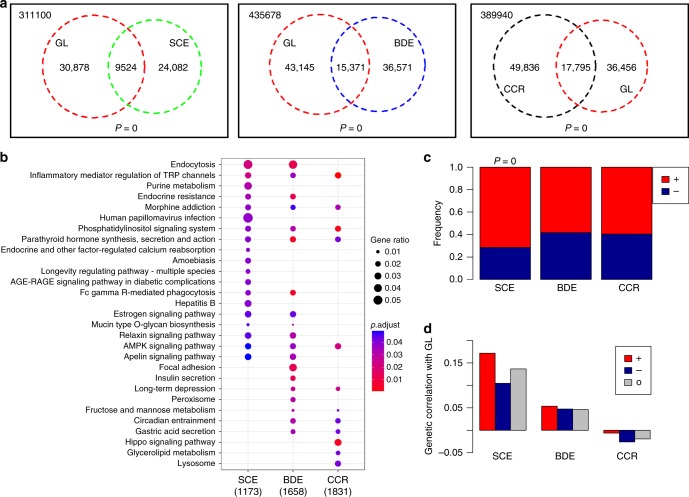
Relationships of sperm methylation alterations associated with gestation length, sire calving ease (SCE), body depth (BDE), cow conception rate (CCR). **a** Intersections between differentially methylated regions (DMRs) of gestation length and DMRs of SCE, BDE and CCR, respectively. *P*-value is calculated using a Fisher-exact test. **b** KEGG pathway functional enrichment of genes that are overlapped with shared DMRs in gestation length & SCE, gestation length & BDE, and gestation length & CCR, respectively. **c** Frequency of shared DMRs with the same (+) or opposite (−) change directions in gestation length & SCE, gestation length & BDE, and gestation length & CCR, respectively. **d** Comparisons of genetic correlations of gestation length & SCE, gestation length & BDE, and gestation length & CCR within their shared DMRs of same (+) or opposite (−) change directions, and over the entire genome (O), respectively

Since sire calving ease (SCE) and body depth (BDE) and cow conception rate (CCR) were genetically correlated with gestation length (Fig. 2a), we hypothesized that they also share the epigenetic architecture in sperm with gestation length. To test this, we further detected DMRs associated with SCE, BDE, and CCR based on the comparisons of high-SCE vs. low-SCE bulls, high BDE vs. low BDE, and high CCR vs. low CCR, respectively. We found that gestation length associated DMRs significantly overlapped with DMRs that were associated with SCE, BDE and CCR, respectively (Fig. 4a). There were 9524, 15,371, and 17,795 DMRs shared in gestation length & SCE, gestation length & BDE, and gestation length & CCR, respectively, which were used as three interesting DMR groups for further comparisons (Fig. 4a). Based on DMRs in these three groups, we defined three groups of genes that overlapped (gene body and promoter) with them. We found that genes in the three groups were commonly and significantly (FDR < 0.05) engaged in parathyroid hormone metabolism, inflammatory mediator regulation of TRP channels, phosphatidylinositol signaling system, and AMPK signaling pathway (Fig. 4b). Notably, for the shared DMRs in gestation length & SCE group, genes were selectively and significantly engaged in the longevity regulating pathway, endocrine-regulated calcium reabsorption and amoebiasis (Fig. 4b), implying their important roles in pregnancy and calving^45–48^. For the shared DMRs in the gestation length & BDE group, genes were significantly engaged in the focal adhesion, insulin secretion and peroxisome (Fig. 4b), suggesting their roles in the regulation of embryo growth^49,50^. For the shared DMRs in the gestation length & CCR group, genes were selectively and significantly engaged in the hippo signaling pathway, glycerolipid metabolism and lysosome (Fig. 4b), indicating their potential roles in the regulation of conception and fertility^51^. In Fig. 4c, for the shared DMRs in the gestation length & SCE group, 71% of the DMRs had the same direction of change for gestation length and SCE (Fig. 4c). In contrast, lower proportions of DMRs (58 and 60%) had the same change direction for gestation length & SCE and gestation length & CCR groups (Fig. 4c). This finding was in line with the genetic evidence that gestation length was more genetically correlated with SCE than with BDE and CCR. Furthermore, we observed that the genetic correlation between SCE and gestation length was higher in their shared DMRs of the same change direction, compared to those of opposite directions or the entire genome background (Fig. 4d). These results further supported that the epigenetic alterations in sperm associated with the genetic architecture underlying gestation length and other dairy traits.

### Multiple evidence implicating *ZNF613* for gestation length

**Fig. 5:**
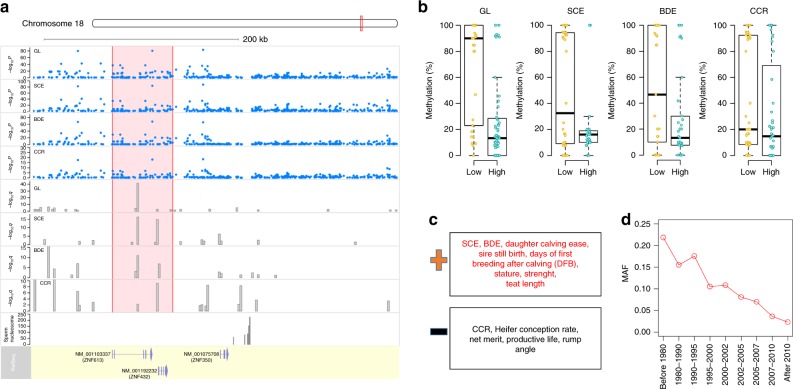
Genetic, epigenetic, and selection evidence implicating the *ZNF613* gene on gestation length. **a** GWAS signals (point-plot) and differentially methylated regions (DMR, bar plot) around *ZNF613* for gestation length, sire calving ease (SCE), body depth (BDE) and cow conception rate (CCR). Sperm-retained nucleosome is also shown below DMRs. **b** Methylation levels of the shared DMR (the second intron of *ZNF613*) in the two compared groups across these four traits. **c** Comparison of signs of variant effects (i.e., *b*, based on signal-marker GWAS) within *ZNF613* between gestation length and 13 dairy traits, for which *ZNF613* is also a candidate gene based on our previous fine mapping analyses. +denotes the same direction, and – denotes the opposite direction. **d** The changes of minor allele frequency (MAF) of the lead variant (chr18:58141989; *P* = 7.97e-84) within *ZNF613* over the years from 1952 to 2012

The most significant QTL of gestation length was on BTA18, which was also significantly associated with SCE, BDE, and CCR (Supplementary Fig. 4). This was consistent with previous studies that reported this QTL to be associated with many fertility and body conformation traits in cattle, including gestation length^21^, stillbirth^52^, calving ability^52–58^, longevity^59^, calf birth weight^19,60^, young stock survival^61^, conformation^54–56,62^, and udder types^55^. However, the high extent of linkage disequilibrium and lack of functional annotation in this QTL region hampered the efforts to determine the causal gene and variants. Here, the fine-mapping analysis in our current and previous studies^63^ identified *ZNF613* as the candidate gene for all of the four traits, including gestation length, SCE, BDE and CCR, which was consistent with previous studies in the Nordic dairy cattle population that proposed *ZNF613* to be associated with calving difficulty and longevity^59,61^. Of note, *ZNF613* had a common DMR on its second intron across all these four traits, and this common DMR was also the top one for gestation length in the corresponding genomic region (Fig. 5a). We further observed that animals with higher gestation length, SCE, BDE, and CCR had lower methylation levels in this particular DMR (Fig. 5b), which implied that the loss of methylation in the second intron of *ZNF613* could be associated with a prolonged gestation, a more difficult calving, a bigger body size, but a higher conception rate. By examining the Bovine Gene Atlas data, we found that *ZNF613* was expressed in 85 out of the 87 tissues, but was highly expressed in the thyroid, placenta above cotlydon, fetal tongue surface, anterior pituitary and hippocampus, suggesting its potential role in the regulation of embryonic development. We further validated that *ZNF613* was highly expressed in the embryonic brain and ovary, intercaruncular tissue (e.g., placenta), thyroid and testes among 174 tissues in sheep^64^ (Supplementary Data 5). Its orthologous gene in mouse, *zfp157*, was highly expressed in the early conceptus and embryo ectoderm, and was associated with phenotypes related to endocrine/exocrine glands, integument and reproductive system (http://www.informatics.jax.org/marker/MGI:1919404). In addition, *ZNF613* was reported to be transcriptionally active at the blastocyst stage before implantation in cattle^43^. We further confirmed this by observing that nucleosomes were retained around *ZNF613* in the cattle mature sperm^65,66^ (Fig. 5b), as sperm-retained nucleosomes package genes for the embryonic development^67^. Thus, it is tempting to propose that *ZNF613* functions at the very early embryonic developmental stage after fertilization, and that its epigenetic marks in sperm play important roles in the regulation of gestation length by influencing the fetal development, as well as that *ZNF613* is related to many fertility and conformation traits potentially due to its effect on gestation length.

Our previous fine-mapping study on 35 complex traits with the same dataset demonstrated that *ZNF613* was also the fine-mapped gene for eight dairy traits (average posterior probability of causality = 0.418), including days of first breeding after calving (DFB), heifer conception rate, net merit, productive life, rump angle, sire still birth, stature, strength and teat length^63^. By comparing the sign of marker effects in *ZNF613* across all these associated traits, we found that gestation length was under the same selection direction with SCE, daughter calving ease, sire still birth, DFB, stature, strength and teat length, while gestation length was under the opposite selection direction with CCR, heifer conception rate, rump angle, net merit and productive life (Fig. 5c). This finding was consistent with the epigenetic evidence that animals with higher methylation levels on the second intron of *ZNF613* had a prolonged gestation, a more difficult calving and a bigger body size; however these animals had a higher conception rate. We found that the minor allele frequency (MAF) of the top associated SNP (chr18:58141989; *P* = 7.97e-84) in *ZNF613* was 0.07 in the current U.S. Holstein population. However, the MAF of this SNP decreased dramatically over the years from 1952 to 2012 (Fig. 5d) due to the strong selection against it, suggesting that current and future selection may quickly remove this undesired variant out of the cattle population. These results demonstrated an example where selection shaped the genetic architecture of complex traits.

## Discussion

The data generated from the dairy industry is a valuable resource that provides a unique opportunity to investigate the paternal contribution to pregnancy related traits such as gestation length. In this study, we combined both genomics and sperm epigenomics data to dissect the paternal effects on gestation. Our GWAS study identified nine QTLs of gestation length passing the genome-wide significance level. Using a Bayesian fine-mapping analysis, we identified 25 candidate genes of gestation length. By investigating multiple sources of functional genomics data, we showed strong evidence supporting *ZNF613* as the candidate gene for the most significant QTL on BTA 18. Despite the evidence we showed in this study, direct functional studies are still needed to validate the candidate genes reported here in the future.

In conclusion, our study is the first to explore the paternal genetic and epigenetic contributions to gestation length in a large cattle population. We demonstrate that paternal effects on gestation length may occur through regulating the embryo development, and the underlying epigenetic architecture of gestation length in sperm correlates with its genetic architecture. Gestation length shares both genetic and epigenetic architecture with other dairy traits of economic importance, such as SCE, BDE and CCR. In addition, we provide genetic, epigenetic, and selection evidence implicating *ZNF613* as the candidate gene for the major QTL on BTA18, indicating that both epigenetic alterations and selection pressures could contribute to the genetic architecture underlying complex traits. Our study also demonstrates the usefulness of integrating multiple layers of biological information to understand the phenotypic variation of complex traits.

## Methods

### Single-marker GWAS

The phenotype and genotype data have been described in previous studies^63,68^. The phenotypes were de-regressed breeding values (predicted transmitting abilities or PTA) with high reliability for 27,214 Holstein bulls, which have been adjusted for known systematic effects including herd, year, season, and parity^10^. The high-density SNP genotypes (*n* = 312 K) of all the individuals were imputed to sequence variants (*n* = 3,148,506) with an imputation accuracy of 96.7%^68^ using reference data from Run 5 of the 1000 Bull Genomes Project^69^. The imputation was conducted with the FindHap software (https://aipl.arsusda.gov/software/findhap). A total of 2,619,418 imputed variants with minor allele frequency (MAF) > 0.01 and Hardy–Weinberg Equilibrium (HWE) test (*P* > 1e-06) were kept for further analyses.

Details of the single-marker GWAS analyses were described in^63^. Briefly, a linear mixed model, implemented in MMAP (https://mmap.github.io/), was employed to test for association of the imputed sequence variants:

$${\boldsymbol{y}} = {\mathrm{\mu }} + {\boldsymbol{X}}b + {\boldsymbol{g}} + {\boldsymbol{e}}$$

where ***y*** is the de-regressed PTA, *μ* is the overall mean, ***X*** is the genotype of a candidate marker (coded as 0, 1, or 2), *b* is the marker effect, ***g*** **~** $N({\bf{0}},\sigma _g^2{\boldsymbol{G}})$ is the polygenic effect accounting for familial relationship and population structure, and ***e*** **~** $N({\bf{0}},\sigma _e^2{\boldsymbol{R}})$ is the residual. ***G*** is the genomic relationship matrix^70^, which was built using HD markers with MAF > 0.01. ***R*** is a diagonal matrix with $R_{ii} = 1/r_i^2 - 1$, where $r_i^2$ is the reliability of phenotype for the *i*th individual. A tested variant with *P* < 1.91e-08 (Bonferroni correction) was considered significant at the genome-wide level.

Besides gestation length, we have also analyzed 35 other dairy traits in a previous GWAS^63^. These 35 traits were clustered into three groups based on their genetic correlations, including 17 body type, 12 reproduction, and 6 production traits. The pair-wise genetic correlations between gestation length and other 35 traits were approximately computed using the Pearson’s correlation of effects (*b*) of all tested variants^71^.

### Gene-level fine-mapping analysis

The genomic regions used for fine-mapping analyses were defined by extending the QTL regions (i.e., the minimal regions covering all significant SNPs) 1 Mb upstream and downstream. We conducted fine-mapping analysis for each of these candidate regions by employing our Bayesian fine-mapping approach (BFMAP) (http://terpconnect.umd.edu/~jiang18/bfmap/)^63^, which follows a similar framework as Huang et al.^72^. BFMAP attempted to determine independent association signals in a genomic region by assessing a posterior probability of causality (PPC) for each variant within this particular region. The PPC of a gene was calculated as the sum of PPCs of all variants that were located within 2 Kb upstream and downstream of the corresponding gene. BFMAP has been shown to have at least equal power as the commonly used fine-mapping approaches^63^, such as PAINTOR^73^ and CAVIARBF^74^.

### Public gene annotation sources and genomic features

We used org.Bt.eg.db v. 3.6.0, reactome.db v. 1.64.0, packages, MeSH v. 1.10.0, which have been implemented in Bioconductor v. 3.7, (https://bioconductor.org/packages/release/data/annotation/html/org.Bt.eg.db.html) to link genes to GO terms, KEGG pathways, Reactome pathways, and MeSH terms. A total of 755 miRNA-target networks were built as previously described^75^. Briefly, 755 cattle miRNAs were obtained from miRbase (http://www.mirbase.org/), The miRmap software^76^ was used to predict the targets of each miRNA, and only the top 25% of predicted targets were considered. For a given trait in the Cattle QTLdb (release 35, April 29 2018, https://www.animalgenome.org/cgi-bin/QTLdb/BT/index), we considered genes that were located inside or in the closest proximity to the QTL regions as associated genes with this particular trait. We excluded traditional QTL mapping results in the QTLdb due to their large QTL regions. In the end, we kept the GO terms (*n* = 889), KEGG pathways (*n* = 225), Reactome pathways (*n* = 804), miRNA-target networks (*n* = 755), and trait-associated gene networks (*n* = 208) that comprised of at least 10 genes as genomic features for further GWAS signal enrichment analyses. For instance, a GO term with ≥10 genes can be considered as a genomic feature.

### GWAS signal enrichment analysis

Because the complex phenotypes being analyzed are highly polygenic or even omnigenic^27,77^, we employed the following sum-based marker-set test approach (http://psoerensen.github.io/qgg/) to determine whether the GWAS signals were enriched in a predefined genomic feature (i.e., a gene list defined using the above annotation sources, including GO terms, KEGG, and Reactome pathways, and miRNA-targets, trait-associated gene networks, and differentially methylation regions). This enrichment analysis is based on all variants in a GWAS study rather than the top variants passing genome-wide significance level. Previous studies have demonstrated that this approach has at least equal power to many commonly used GWAS signal enrichment methods in human^78^, *Drosophila melanogaster*^79^ and livestock species^80–82^, particularly in highly polygenic phenotypes. The sum-based statistics can be expressed as

$${\mathrm{T}}_{sum} = \mathop {\sum }\limits_{i = 1}^{m_f} b^2$$

where m_*f*_ is the number of genomic variants in a pre-defined genomic feature, and *b* is the variant effect. Here, SNPs located in different genes within a genomic feature (e.g., a biological pathway) were often not in linkage disequilibrium (LD). This approach is similar to the popular LD score regression in human studies^83^, and it controls LD patterns among variants and variant-set sizes through employing the following genotype cyclical permutation strategy^78,79^. In brief, we first ordered the test statistics (i.e., *b*^2^) for all variants according to their chromosome positions (i.e., $b_1^2$, $b_2^2$, ⋯ $b_{m - 1}^2$, $b_m^2$). We then randomly chose one test statistic (i.e., $b_k^2$) from this vector as the first, and shifted the remaining test statistics to new locations, while retained their original orders (i.e., $b_k^2$, $b_{k + 1}^2$, ⋯ $b_{m - 1}^2$, $b_m^2$, $b_1^2$⋯ $b_{k - 1}^2$) to maintain the correlation patterns among variants. We calculated a new summary statistic for the tested genomic feature using its original genomic positions. We repeated this permutation procedure 10,000 times for each genomic feature being tested, and obtained an empirical *P*-value by using a one-tailed test of the proportion of random summary statistics greater than that observed.

### Sperm WGBS data and bioinformatics analyses

No animal experiments were conducted in this study, and ethics committee approval was thus not required. All sperm methylation data were generated in previous studies, and references were provided where animal data were used.

The sperm whole-genome bisulfite sequencing (WGBS) data were generated in previous studies^66^. All the semen samples used in this study were collected from bulls by an artificial insemination company using a standardized procedure with artificial vaginas. Briefly, one semen straw (0.5 ml) often contains 10–40 million sperm, and is transported and stored in a liquid nitrogen tank. After thawing a semen straw, PBS buffer was used to wash away the extender for three times by mild centrifugations. Visual examination of washed sperm samples was conducted under a microscope, and over 90% of sperm cells were found morphologically normal. At first, eight semen straws were sampled from eight representative Holstein bulls, among which four animals have high PTA of gestation length and the other four with low PTA of gestation length (Supplementary Table 1). For other traits such as SCE, BDE, and CCR, we also collected six semen samples with two groups (i.e., high PTA *vs*. low PTA) for each trait, and each group then had three biological replicates. In total, all of these sperm samples were from 18 fertile, age-matched and representative animals, because several animals had extreme values for multiple traits. The reliabilities of PTA for all of the 18 animals were greater than 0.97, 0.93, 0.87 and 0.83 for gestation length, SCE, BDE and CCR, respectively. Genomic DNA was isolated using QIAamp DNA Mini Kit protocol (QIAGEN, Valencia, CA, USA). The quality of isolated DNA was evaluated using the 2100 Bioanalyzer (Agilent Technologies, Santa Clara, CA, USA). The sequence libraries were constructed using all of the qualified genomic DNA^66^, and were then sequenced using HiSeq X 10 (Illumina, San Diego, CA, USA) with a 150 bp paired-end technology.

FastQC v0.11.2 (https://www.bioinformatics.babraham.ac.uk/projects/fastqc/) and Trim Galore v 0.4.0 (https://www.bioinformatics.babraham.ac.uk/projects/trim_galore/) were employed to check and clean the raw data, respectively^66^. Generally, adapters and reads with low quality (Q < 20) or shorter than 20 bp were removed. All the cleaned data were mapped to the cattle reference genome (UMD 3.1) using bowtie2^84^ with an average mapping rate of 69.23% (ranging from 53.10 to 78.90%). The total number of mapped reads per sample ranged from 134,984,436 to 227,534,395 with an average of 171,982,028. Bismark software^85^ was applied to extract methylcytosine information. Only the loci that were covered by at least 10 clean reads were kept for further analyses. More details have been described previously^66^.

Because methylation alterations that are associated with complex traits often exhibit spatial correlation patterns^86^, DMR (differentially methylated region) instead of DMC (differentially methylated cytosine) were determined by using methylKit^87^. The entire genome was first tiled into windows of 2000 bp in length and 2000 bp step-size, and then methylation levels on those tiles were summarized. A logistic regression model implemented in the *calculateDiffMeth* function was employed to detect DMR: information from each sample is specified (the number of methylated Cs and number of unmethylated Cs at a given region), and a logistic regression test is applied to compare fraction of methylated Cs across the two groups under comparison. *P*-values were calculated through comparing the model fitness of alternative models to the null model, and were corrected to *q*-values for multiple testing using the SLIM method^88^. Since methylation alterations associated with complex phenotypes are generally very weak^86,89^, the absolute value of difference in methylation >5% and different *q* cutoffs (i.e., 0.05, 0.01, 1e-5, 1e-8, and 1e-10) were used to define DMR for the downstream analyses.

### DMR-set enrichment analysis

The following count-based approach was used to test whether a Reactome pathway was enriched for DMR,

2 $${\mathrm{T}}_{count} = {\sum}_{i = 1}^{m_f} {\mathrm{I}}\;(q_i \, < \, q_0),$$

where *m*_*f*_ is the total number of 2000 bp-tiles being tested that were overlapped (at least 1 bp) with genes in a pathway, *q*_*i*_ is the *q* value for the *i*th tested tile, *q*_*0*_ is an arbitrarily chosen threshold, and I is an indicator function that takes value 1 when $q_i < q_0$, and value zero otherwise. Here *q*_0_ = 0.01 was used tentatively, and gene regions were extended 10 Kb upstream and downstream to cover potential regulatory regions. Under the null hypothesis (i.e., DMR are distributed in the genome randomly), T_*count*_ was assumed to follow a hypergeometric distribution: T_*count*_ ~ Hyper(m, m_g_, m_f_) where m is the total number of tiles being tested in the entire genome, m_g_ is the total number of DMR detected in the entire genome, and m_*f*_ is the number of tiles being tested in a pathway. The null hypothesis (i.e., no enrichment) will be rejected when the adjusted *P-*value is less than 0.05. Here, a tile was considered belonging to a pathway if the tile intersected any gene (±10 Kb up- and down-stream) in the particular pathway.

Functional enrichment analyses for gene lists in DMRs were conducted using the R package clusterProfiler^90^, where a hypergeometric test was employed using the current KEGG database. *P*-values were adjusted for multiple testing using the FDR method^91^, and FDR < 0.05 was considered as significant.

### Reporting summary

Further information on experimental design is available in the Nature Research Reporting Summary linked to this article.

## Supplementary information

Supplementary Information

Description of Additional Supplementary Files

Reporting Summary

Supplementary Data 1

Supplementary Data 2

Supplementary Data 3

Supplementary Data 4

Supplementary Data 5

## Data Availability

All the 18 cattle sperm methylomes have been submitted to NCBI with accession number GSE119263. All genomic annotation files of cattle (UMD 3.1.1) are available for download from Ensembl database (https://uswest.ensembl.org/index.html). The authors confirm that the original genotype data are owned by the Council on Dairy Cattle Breeding (CDCB). A request to CDCB is necessary for getting data on research, which may be sent to: João Dürr, CDCB Chief Executive Officer (joao.durr@cdcb.us). All other data have been shown in the manuscript and supplementary data.
