# Supplementary material for: Genetic and epigenetic architecture of paternal origin contribute to gestation length in cattle
Source: Commun Biol. 2019 Mar 14;2:100. doi: 10.1038/s42003-019-0341-6 (PMC6418173; doi:10.1038/s42003-019-0341-6)
Supplement: Supplementary file 1 — Supplementary Information [file 42003_2019_341_MOESM1_ESM.pdf]

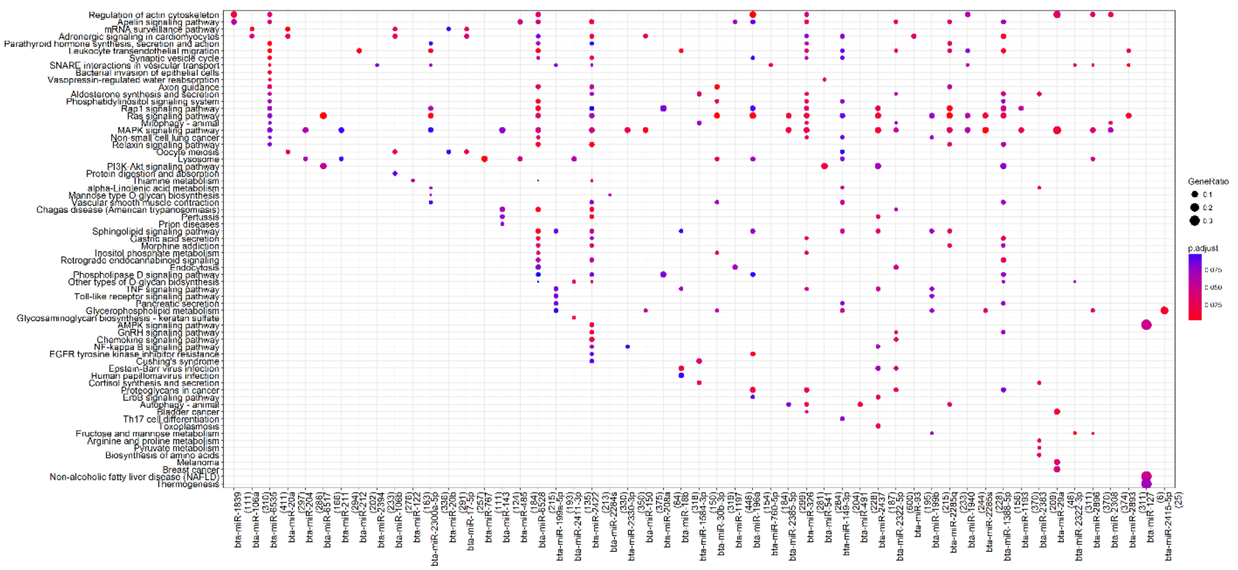

**Supplementary Figure 1. The KEGG pathway enrichment analysis for miRNA-targets that were significantly enriched with GWAS signals of gestation length.** p.adjust is FDR based on a hypergeometric test, implemented in the R package clusterProfiler. GeneRatio is k/n, where k is the size of the overlap of the targets of a miRNA with a specific KEGG pathway, n is the size of the overlap of the targets of a miRNA with all genes annotated in the KEGG database.

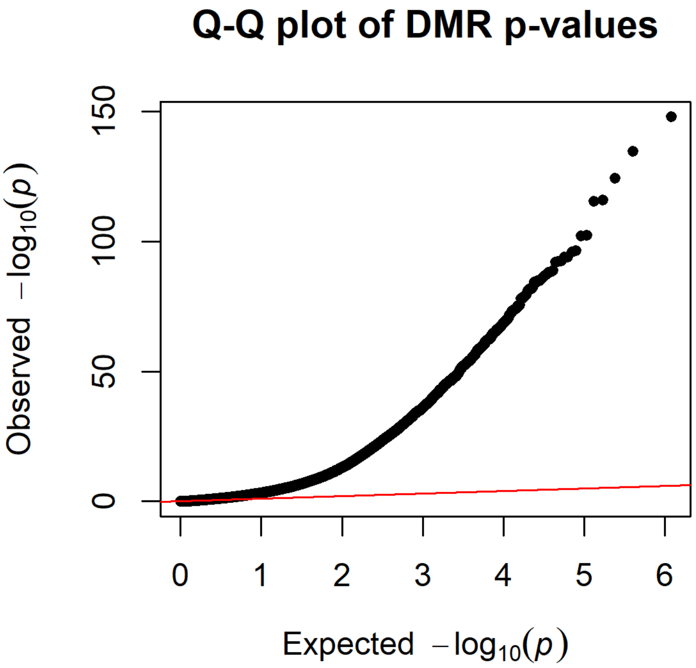


**Supplementary Figure 2. The Q-Q plot of the differential methylation analysis in sperm for gestation length**

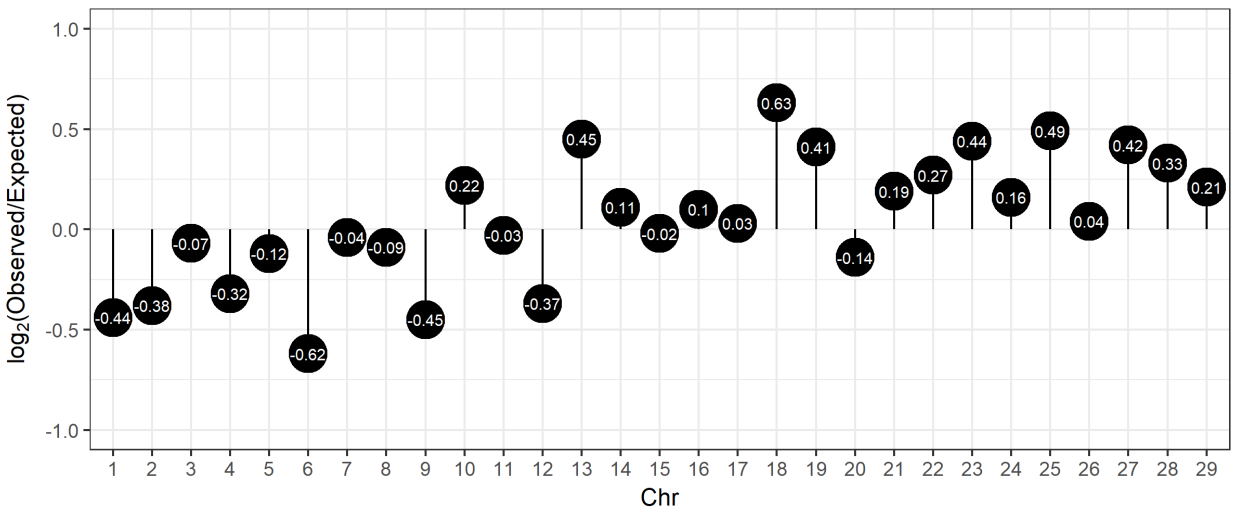


**Supplementary Figure 3. The enrichment of significantly differentially methylated regions (DMR) across all the 29 autosomes in cattle.** The value is calculated as Observed (O)/Expected (E), where O is the percentage of DMR length over all the tested methylation regions in a chromosome, and E is the percentage of the particular chromosome length over the entire.

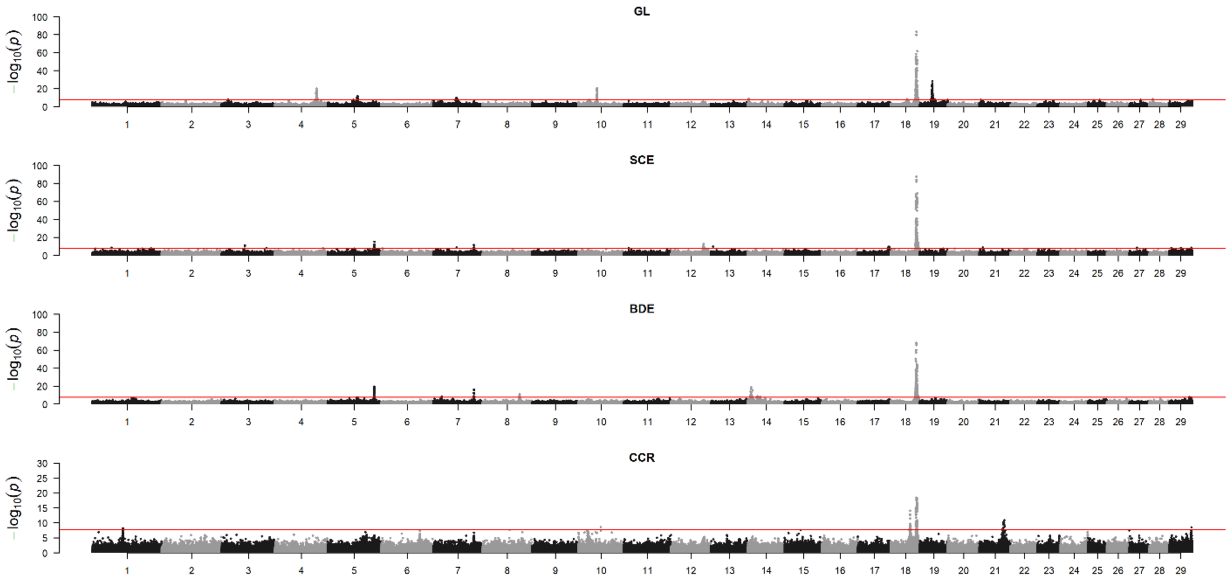


**Supplementary Figure 4. The Manhattan plots of genome-wide association analysis for gestation length (GL), sire calving ease (SCE) and body depth (BDE), and cow conception rate (CCR)**.

**Supplementary Table 1. Phenotypes (i.e., PTA for gestation length, sire calving ease, body depth, and cow conception rate) and their reliabilities of the sperm DNA methylation samples.**

| Gestation Length | | | Sire Calving Ease | | | Body Depth | | | Cow Conception Rate | | |
|---|---|---|---|---|---|---|---|---|---|---|---|
| PTA | Reliability | Group | PTA | Reliability | Group | PTA | Reliability | Group | PTA | Reliability | Group |
| 2.7 | 0.97 | High | 10.6 | 0.90 | High | 1.99 | 0.99 | High | 7 | 0.98 | High |
| 2.6 | 0.99 | High | 9.4 | 0.93 | High | 0.87 | 0.99 | High | 5.8 | 0.83 | High |
| 1.5 | 0.97 | High | 9 | 0.98 | High | 0.86 | 0.90 | High | 5.2 | 0.87 | High |
| 1.5 | 0.99 | High | 5.5 | 0.99 | Low | -1.05 | 0.87 | Low | 0.4 | 0.87 | Low |
| -2 | 0.97 | Low | 5.5 | 0.95 | Low | -1.08 | 0.99 | Low | -2.2 | 0.93 | Low |
| -2.5 | 0.97 | Low | 4.5 | 0.99 | Low | -1.34 | 0.99 | Low | -3.8 | 0.99 | Low |
| -2.5 | 0.99 | Low | | | | | | | | | |
| -3.2 | 0.99 | Low | | | | | | | | | |
