# Supplementary material for: Genetic and epigenetic architecture of paternal origin contribute to gestation length in cattle
Source: Commun Biol. 2019 Mar 14;2:100. doi: 10.1038/s42003-019-0341-6 (PMC6418173; doi:10.1038/s42003-019-0341-6)
Supplement: Supplementary file 2 — Description of Additional Supplementary Files [file 42003_2019_341_MOESM2_ESM.pdf]

**Description of Additional Supplementary Files**

**File Name**: Supplementary Data 1

**Description**: The fine-mapping results for significant QTL regions in genome-wide association analysis. A Bayes fine-mapping method was used for fine-mapping, details in Methods. The Variants List file contains all the informations for the individual variants with the normed posterior probability of causality (PPC) > 0.003. The Gene Information contains all the genes that include the variants in the Variants List, the PPC of each gene is the sum of PPC of variants inside it. The GO annotation file contains the GO terms that the fine-mapped genes were involved in.

**File Name**: Supplementary Data 2

**Description**: The GWAS signal enrichment results for gestation length across six public annotation databases. The data were generated from the GWAS signal enrichment analysis, and were used to understand the biological basis of gestation length.

**File Name**: Supplementary Data 3

**Description**: The enrichment analysis for differentially methylated regions (DMR) of gestation length using Reactome database. The data were generated by the DMR enrichment analysis (see Methods), and were used to study the biological implication of DMRs of gestation length. Both file is the results for all the DMRs. The Loss and Gain contain results for DMRs that loss and gain in animals with higher gestation length, respectively.

**File Name**: Supplementary Data 4

**Description**: The summary information for fine-mapping gene overlapping with differentially methylated regions (DMR) of gestation length. The data were generated by overlapping DMR and finemapping genes, in order to provide more evidence to detect causal genes for gestation length

**File Name**: Supplementary Data 5

**Description**: The expression levels of ZNF613 across the 173 tissues in sheep. The data were collected from previous study (sheep atlas; https://omictools.com/sheep-atlas-tool), in order to explore the tissue-specific expression pattern of ZNF613.
